# Advanced glycation end-products are associated with diabetic neuropathy in young adults with type 1 diabetes

**DOI:** 10.3389/fendo.2022.891442

**Published:** 2022-10-11

**Authors:** Elaf Al-Saoudi, Marie M. B. Christensen, Peter Nawroth, Thomas Fleming, Eva E. Hommel, Marit E. Jørgensen, Jesper Fleischer, Christian S. Hansen

**Affiliations:** ^1^ Department of Complication Research , Steno Diabetes Center Copenhagen, Herlev, Denmark; ^2^ Department of Clinical Epidemiology, Steno Diabetes Center Copenhagen, Herlev, Denmark; ^3^ Department of Medicine I and Clinical Chemistry, University Hospital of Heidelberg, Heidelberg, Germany; ^4^ German Center for Diabetes Research (DZD), Neuherberg, Germany; ^5^ Joint Heidelberg-Institute for Diabetes and Cancer (IDC) Translational Diabetes Programm, Helmholtz-Zentrum, Heidelberg, Germany; ^6^ Steno Diabetes Center Copenhagen, Herlev, Denmark; ^7^ Steno Diabetes Center Greenland, Nuuk, Greenland; ^8^ Steno Diabetes Center Aarhus, Aarhus, Denmark

**Keywords:** advanced-glycation end-products, AGEs, type 1 diabetes, cardiovascular autonomic neuropathy, peripheral neuropathy, distal symmetric polyneuropathy

## Abstract

**Aims/hypothesis:**

Advanced glycation end-products (AGEs) may contribute to the development of diabetic neuropathy. In young adults with type 1 diabetes, we aimed to investigate the association between AGEs and cardiovascular autonomic neuropathy (CAN) and distal symmetric polyneuropathy (DSPN).

**Methods:**

This cross-sectional study comprised 151 young adults. CAN was assessed by cardiovascular autonomic reflex tests; lying-to-standing test, deep breathing test (E/I), Valsalva manoeuvre, and heart rate variability indices; and the mean square of the sum of the squares of differences between consecutive R-R intervals and standard deviation of normal-to-normal intervals (SDNN), high- (HF) and low-frequency (LF) power, total frequency power, and the LF/HF ratio. DSPN was assessed by light touch, pain and vibration perception threshold (VPT), neuropathy questionnaires, and objective measures. AGEs were analysed in four groups using z-scores adjusted for relevant confounders and multiple testing: i) “glycolytic dysfunction”, ii) “lipid peroxidation”, iii) “oxidative stress”, and iv) “glucotoxicity”.

**Results:**

A higher z-score of “glycolytic dysfunction” was associated with higher VPT (4.14% (95% CI 1.31; 7.04), p = 0.004) and E/I (0.03% (95% CI 0.01; 0.05), p = 0.005), “lipid peroxidation” was associated with higher LF/HF ratio (37.72% (95% CI 1.12; 87.57), p = 0.044), and “glucotoxicity” was associated with lower SDNN (−4.20% (95% CI −8.1416; −0.0896), p = 0.047). No significance remained after adjustment for multiple testing.

**Conclusions/interpretations:**

In young adults with type 1 diabetes, increased levels of AGEs involving different metabolic pathways were associated with several measures of CAN and DSPN, suggesting that AGEs may play a diverse role in the pathogeneses of diabetic neuropathy.

## Introduction

Diabetic neuropathy is a leading cause of morbidity and mortality in both type 1 and type 2 diabetes (1, 2). The progressive nature with insidious onset and varying symptoms and clinical manifestations often lead to delayed diagnosis with severe and irreversible symptoms (1). Cardiovascular autonomic neuropathy (CAN) and distal symmetric polyneuropathy (DSPN) are the most prevalent types of diabetic neuropathy with varying prevalence reaching up to 35% and 41%, respectively, in adults with type 1 diabetes (3–7). Previous studies have identified a high prevalence of definite and subclinical manifestations of diabetic neuropathy in young adults with type 1 diabetes (8–12). This suggests that early screening in adolescence may detect early stages of CAN and DSPN, where prevention of progression may still be possible. Although diagnostic methods for both types of neuropathy are available, it remains unknown which underlying mechanisms are involved and lead to painful versus insensate symptoms (1).

Hyperglycaemia and metabolic derangements are suggested to play an important role in the progression of neurological complications (1, 13). One of the pathogenic pathways described is the formation of reactive metabolites leading to increased levels of advanced glycation end-products (AGEs) as a direct consequence of hyperglycaemia and lipid peroxidation (13–15). The accumulation of AGEs tends to alter protein function and thereby the structure of the nerve tissue, contributing to the development of neuropathy (15).

In the serum of diabetic patients, AGEs are elevated alongside levels of their main precursors: glucose and the dicarbonyls methylglyoxal (MG), glyoxal, and 3-deoxyglucosone (3-DG) (13, 16).

While studies regarding the dicarbonyls 3-DG and glyoxal and their corresponding AGEs are scarce (13, 17, 18), MG and MG-derived AGEs (i.e., methylglyoxal-derived hydroimidazolone 1 (MG-H1)) have been associated with the progression of diabetic neuropathy, retinopathy, and nephropathy in type 1 diabetes. Likewise, increased plasma levels of MG and MG-H1 have been associated with diabetic painful peripheral neuropathy in both type 1 and type 2 diabetes (16, 17).

Also, the glucose-derived AGEs, fructose lysine (FL) and glucosepane, have been identified as strong predictors of diabetic neuropathy, supporting that high glucose levels may generally associate with the risk of complications and therefore have been targeted for treatment (1, 17, 19). However, good glycaemic control does not necessarily prevent the progression of diabetic neuropathy (20). Some studies have found increased levels of MG in both type 1 and type 2 diabetes independent of blood glucose levels, suggesting that factors other than hyperglycaemia are involved in late diabetes complications (14).

Although several AGEs have been associated with diabetic neuropathy, identifying specific pathways involved in the pathogeneses of neuropathy is missing. Furthermore, diabetic neuropathy represents a collection of syndromes, and therefore, subtypes of neuropathy symptoms may be related to different dysfunctional pathways.

In this cross-sectional study, we aim to define the metabolic pathways leading to the formation or accumulation of AGEs and relate them to measures of CAN and DSPN and thereby to specify potential pathways associated with distinct neuropathy signs and/or symptoms. This will be performed in a Danish population of young adults with type 1 diabetes using objective sensitive age-matched measuring methods to detect even early signs of neuropathy.

## Methods

### Study design and study population

The study was designed as a cross-sectional observational study and has previously been described in detail (8). In brief, inclusion criteria were type 1 diabetes and age >17 and <25 years. The patients were recruited from the outpatient clinic at Steno Diabetes Center Copenhagen, Gentofte, Denmark. Of the 340 eligible participants who received an invitation to participate, 156 were accepted. Five participants were excluded due to missing biochemical measures, leaving 151 included in the study. Ethical approval was obtained from the Danish Research Ethics Committee (ID No. H-15006967), and written informed consent was obtained from all patients prior to examination.

### Measures of diabetic neuropathy

Measures of DSPN were assessed and categorized according to recommendations made by the Toronto Diabetic Neuropathy Expert Group (2) and included the following:

-Symptoms of DSPN were assessed by the questionnaires Brief Pain Inventory (BPI) and Michigan Neuropathy Screening Instrument (MNSI). Diabetic neuropathy was, in the BPI questionnaire, defined as the presence of pain in both legs and/or both arms peripherally and, in the MNSI questionnaire, as a score of ≥7 (21).
-Signs of DSPN were assessed by established measures including light touch perception using a 10-g monofilament, pain perception using a pinprick device, and vibration perception threshold (VPT) determined by using a biothesiometer. To assess abnormal VPT tests, age-stratified perception thresholds were used (12). The tests were pathological if the results were abnormal bilaterally.
-Objective measuring methods using the non-invasive device Sudoscan™ to test for electrochemical skin conductance (ESC) on hands and feet and the handheld NC-stat^®^ to measure sural nerve conduction velocity (SNCV) and sural nerve amplitude potential (SNAP).

To identify abnormal results for the ESC test and measures of SNCV and SNAP, age- and gender-stratified thresholds (22) and age- and height-stratified thresholds were applied, respectively. When abnormalities in SNCV, SNAP, or both bilaterally were found, the measure of sural nerve conduction (SNC) was used.

The definition of DSPN was stratified into four categories: “possible DSPN” if the patient had symptoms or signs according to the abovementioned measures, “probable DSPN” in the presence of symptoms and signs, and “confirmed DSPN” if the patient had a combination of either abnormal test for SNC or ESC and the presence of either symptoms or signs. The presence of abnormal SNC or ESC without symptoms or signs was defined as “subclinical DSPN”.

CAN was evaluated by three standard cardiovascular autonomic reflex tests (CARTs) and measures of 5-min resting heart rate variability (HRV) using the device Vagus™ and performed in a quiet examination room in the afternoon.

After 5 min of supine rest, HRV measures were analysed in time and frequency domain from 5-min resting heart rate (HR). Time domain included the mean square of the sum of the squares of differences between consecutive R-R intervals (RMSSD) and standard deviation of normal-to-normal intervals (SDNN). Frequency-domain analyses included low-frequency power band (LF), high-frequency power band (HF), total frequency power (Total), and the ratio of low-frequency power/high-frequency power (LF/HF ratio).

After the 5-min resting HRV test, the three CARTs for diagnosing CAN were performed and included the lying-to-standing test (30:15), the deep breathing test (E/I), and the Valsalva manoeuvre (23). The participants were asked to restrain from vigorous exercise 24 h prior to the examination and intake of caffeine on the specific day of examination.

The diagnosis of “early CAN” and “definite CAN” was given according to age-stratified thresholds (3) if one of the three CARTs was abnormal and if two or three tests were abnormal, respectively.

### Dicarbonyls and advanced glycation end-products

The plasma levels of AGEs were grouped according to the main pathways of AGE formation (13) that proceed *via* glucose and the reactive dicarbonyls MG, glyoxal, and 3-DG:

- “Glycolytic dysfunction” includes the AGEs derived from methylglyoxal: MG-H1, *N* ^ε^-(carboxyethyl)-lysine (CEL), methylglyoxal-lysine dimers (MOLD), and argpyrimidine. As methylglyoxal comes from glycolysis, increased methylglyoxal and methylglyoxal-derived AGEs will represent a dysregulation of glycolysis.
- “Lipid peroxidation” includes the glyoxal-derived AGEs: glyoxal-derived hydroimidazolone 1 (G-H1) and *N* ^ε^-(carboxymethyl)-lysine (CML). Glyoxal and glyoxal-derived AGEs derive from the degradation of lipids, and therefore increased levels of glyoxal and glyoxal-derived AGEs will represent increased oxidation of lipids.
- “Oxidative stress” includes AGEs generated from the direct interaction of reactive oxygen species (ROS) with methionine: methionine sulfoxide.
- “Glucotoxicity” includes AGEs and dicarbonyls derived directly from the modification of amino acids by glucose: fructose lysine (FL), glucosepane, and 3-DG.
- “Dicarbonyls”: MG, glyoxal, and 3-DG. The dicarbonyls were also analysed separately in a fifth group:

### Clinical data collection

As described previously, data on medication were extracted from hospital electronic records, and lifestyle factors including smoking status and weekly amount of exercise were recorded in a questionnaire filled in by the patients.

Blood pressure and heart rate were measured after 10 min of rest using automated oscillometric blood pressure recorders and calculated as the mean of three consecutive measures performed with intervals of 1 min. Height and weight were measured with clothes on and without shoes using a fixed rigid stadiometer and an electronic scale, respectively.

### Biochemical measures

HbA_1c_, serum total cholesterol, serum high-density lipoprotein (HDL) cholesterol, serum triglycerides, and plasma creatinine were measured from non-fasting venous blood samples and collected on the examination day. HbA_1c_ was analysed by high-performance liquid chromatography on a Tosoh G7 (Tosoh Corporation, Tokyo, Japan). Triglycerides, HDL, and total cholesterol were analysed by standard enzymatic colorimetry techniques on a Vitros 5600 (Ortho Clinical Diagnostics, Illkirch-Graffenstaden, France). Serum LDL cholesterol was calculated using the Friedewald equation.

All biochemical measures were analysed in the laboratory at Steno Diabetes Center, Denmark, except for the plasma samples used for the analysis of AGEs and dicarbonyls. The samples were stored at −80°C at Steno Diabetes Center and transferred on dry ice to the laboratory at the University of Heidelberg, Germany, for analysis.

### Assessment of glycaemic variability indices

As described previously, the continuous glucose monitoring (CGM) sensor Enlite™ (Medtronic, Northridge, CA, USA) was inserted into the subcutaneous tissue of the abdomen or the upper arm and was worn for 5 days (24). The capillary finger blood glucose was monitored four times daily for calibration. To generate data from the sensors, the software Medtronic Carelink™ iPro™ was used. To quantify glycaemic variability, coefficient of variation (CV), standard deviation (SD), continuous overall net glycaemic action, and mean amplitude of glucose excursions were used. Time spent in hypoglycaemia (<3.0 mmol/L), euglycaemia (≥3.0; ¾10.0 mmol/L), and hyperglycaemia (>10.0 mmol/L) were calculated and presented in minutes and percentage.

### Measurement of dicarbonyls

The dicarbonyl content in plasma was determined by isotope dilution and tandem mass spectroscopy, following derivatization with 1,2-diaminobenzene. Briefly, 20 µl of serum was precipitated by addition of 10 µl of ice-cold 20% (wt/vol) trichloroacetic acid in 0.9% (wt/vol) sodium chloride (20 µl) and water (40 µl) (25). An aliquot (5 µl) of the internal standard (400 nM of [^13^C_2_]-Glyoxal, [^13^C_3_]-methylglyoxal, and [^13^C_6_]-3-deoxyglucosone) was then added, and the samples vortex-mixed. Following centrifugation (14,000 rpm; 5 min at 4°C), 35 µl of the supernatant was transferred to high-resolution mass spectrometry (HPLC) vials containing a 200-µl glass interest. An aliquot (5 µl) of 3% sodium azide (wt/vol) was then added to each sample followed by 10 µl of 0.5 mM of DB in 200 mM of HCl containing 0.5 mM of diethylenetriaminepentaacetic acid (DETAPAC) in water. The samples were then incubated for 4 h at room temperature, protected from the light. Samples were then analysed by liquid chromatography–tandem mass spectrometry (LC-MS/MS) using an ACQUITY™ ultra-high-performance liquid chromatography system with a Xevo-TQS LC-MS/MS mass spectrometer (Waters, Manchester, UK). The column was a Waters BEH C18 (100 × 2.1 mm) and a guard column (5 × 2.1 mm). The mobile phase was 0.1% formic acid in water with a linear gradient of 0%–100% 0.1% formic acid in 50% acetonitrile:water over 0–10 min; the flow rate was 0.2 ml/min, and the column temperature was 5°C. The capillary voltage was 0.5 kV; the cone voltage was 20 V; the interscan delay time was 100 ms; the source and desolvation gas temperatures were 150°C and 350°C, respectively; the cone gas and desolvation gas flows were 150 and 800 L/h, respectively. Mass transitions (parent ion > fragment ion; collision energy), retention time, limit of detection, and recoveries were as follows: 3-deoxyglucosone, 235.0 > 199.1; 14 eV, 4.09 min, 0.36 pmol, 95%, glyoxal, 130.9 > 77.1; 22 eV, 5.28 min, 1.15 pmol, 97%, methylglyoxal, 145.0 > 77.1; 24 eV, 5.93 min, 0.52 pmol, 98%. Acquisition and quantification were completed with MassLynx 4.1 and TargetLynx 2.7 (Waters^®^).

### Measurement of protein-free advanced glycation end-products

Protein-free AGEs in the plasma were determined by isotope dilution and tandem mass spectroscopy, as previously described (26). Briefly, an aliquot of plasma (20 µl) was diluted to 500 µl with water and filtered by microspin ultrafiltration (10 kDa cutoff) at 14,000 rpm for 30 min at 4°C. The ultrafiltrate was then retained for the free adduct analysis. An aliquot of the sample (ca. 30 µl) was spiked with an equal volume of 0.2% trifluoroacetic acid (TFA) in water containing the isotopic standards (5–25 pmol). Normal and isotopic standards were either purchased (Cambridge Isotope, Polypeptide Laboratories, Iris Biotech) or prepared in-house, as described previously. Samples were then analysed by LC-MS/MS using an ACQUITY ultra-high-performance liquid chromatography system with a Xevo-TQS LC-MS/MS spectrometer (Waters). Two 5-µm Hypercarb™ columns (Thermo Scientific, Waltham, MA, USA) in series were used: 2.1 × 50 mm, fitted with a 5 × 2.1 mm pre-column, and 2.1 × 250 mm. The mobile phases were 0.1% TFA in water and 0.1% TFA in 50% water. The column temperature and flow rates were 30°C and 0.2 ml/min, respectively. Analytes were eluted using a two-step gradient, and the columns were washed after each sample with 0.1% TFA in 50% tetrahydrofuran (THF), as described previously (26). AGEs, including oxidation and nitration markers, were detected by electrospray positive ionization with multiple reaction monitoring (MRM). The ionization source temperature was 150°C, and the desolvation temperature was 500°C. The cone gas and desolvation gas flows were 150 and 1,000, L/h, respectively. The capillary voltage was 0.5 kV. Molecular ion and fragment ion masses, as well as cone voltage and collision energy, were optimized to ±0.1 Da and ±1 eV for MRM detection of the analytes. Acquisition and quantification were completed with MassLynx 4.1 and TargetLynx 2.7 (Waters^®^).

### Statistical analysis

Patient characteristics are represented as means with SD for normally distributed continuous data, as medians with interquartile range (IQR) for skewed distributed data, and as numbers (%) for categorical data.

A standardized z-score was calculated for each of the following groups of AGEs and dicarbonyls and was examined as a determinant for neuropathy: i) “glycolytic dysfunction” (MG-H1, CEL, and MOLD), ii) “lipid peroxidation” (G-H1 and CML), iii) “oxidative stress” (methionine sulfoxide), iv) “glucotoxicity” (FL, glucosepane, and 3-DG), and v) “dicarbonyls” (MG, glyoxal, and 3-DG).

Outcomes were all continuous, assessed using linear regression analyses, and presented as estimates of a one-unit difference in z-score with 95% CI.

Three models of adjustments were applied: model 1 was adjusted for age and gender, model 2 was adjusted as model 1 + for diabetes duration and HbA_1c_, and model 3 was adjusted as model 2 + for current smoking, total cholesterol, triglycerides, systolic blood pressure, and the use of beta blockers.

All analyses used two-sided p = 0.05 as statistically significant and were adjusted for multiple tests by the Benjamini–Hochberg procedure (27).

Statistical analyses were performed in SAS 9.4 (SAS Institute, Cary, NC, USA).

## Results

### Patient characteristics

The study population included 151 patients (42.4% men) with a mean age of 22 years (SD 1.6). The mean diabetes duration was 11 years (SD 5.1), and HbA_1c_ was 66.5 mmol/mol (IQR 58.0; 77.0). Seventy-two patients (47.7%) were treated with continuous subcutaneous insulin infusion (CSII), and the rest were treated with multiple-dose injections (MDIs). All patients were on insulin (**Table 1**).

**Table 1:** Characteristics of the study population.

Clinical characteristics	Mean (SD)/median (IQR)/N (%)
N	151
Age (years)	22 (1.6)
Male (%)	64 (42.4)
Diabetes duration (years)	11.3 (5.1)
CSII treatment (%)	72 (47.7)
Current smoker (%)	33 (21.9)
Systolic blood pressure (mmHg)	125.3 (11.5)
HbA_1c_ (mmol/mol)	67.0 (58.0;77.0)
HbA_1c_ (%)	8.3 (7.4; 9.2)
Cholesterol (mmol/L)	4.5 (1.2)
Triglycerides (mmol/L)	1.1 (0.8; 1.6)
LDL (mmol/L)	2.6 (0.9)
Urine albumin/creatinine ratio (mg/g)	6 (4.0; 14.0)
eGFR (ml/min/1.73 m^3^)	123.9 (116.6; 129.3)
*Medication*	
Insulin (%)	151 (100)
Metformin (%)	1 (0.7)
Other glucose-lowering drugs (%)	1 (0.7)
Antihypertensive treatment (%)	6 (4.0)
Beta-blocker treatment n (%)	2 (1.3)
Lipid-lowering treatment (%)	2 (1.3)
Psychotropics (%)	6 (4.0)
*CAN measures*	
Definite CAN (%)	13 (8.7)
Early CAN (%)	42 (28.4)
*DSPN measures*	
Confirmed DSPN (%)	4 (2.7)
Subclinical DSPN (%)	82 (54.3)
Probable DSPN (%)	0 (0)
Possible DSPN (%)	5 (3.3)
*BPI questionnaire*	
Painful neuropathy (% answered yes)	3 (2.0)
*MNSI questionnaire*	
MNSI neuropathy (score ≥ 7 points)	0 (0)
*Serum dicarbonyls levels*	
Methylglyoxal (nmol/L)	116.3 (111.3; 127.6)
Glyoxal (nmol/L)	215.7 (186.1; 253.4)
3-Deoxyglucosone (nmol/L)	57.8 (54.3; 62.6)

Data are given in means (SD), medians (IQR), or proportions %.

SD, standard deviation; IQR, interquartile range; CSII, continuous subcutaneous insulin infusion; LDL, low-density lipoproteins; eGFR, estimated glomerular filtration rate; CAN, cardiovascular autonomic neuropathy; DSPN, distal symmetric polyneuropathy; BPI, Brief Pain Inventory; MNSI, Michigan Neuropathy Screening Instrument.

Previously, we investigated the association between glycaemic variability and diabetic neuropathy in the same study population (24). Of the included patients, 133 had CGM. During the 5-day CGM monitoring, mean (SD) CV was 40% (10), median (IQR) SD was 3.9 mmol/L, and time spent on hypoglycaemia was 35 min (0, 120)/1.0% [0.0; 4.0].

The prevalence of subclinical and possible DSPN was 54.3% and 3.3%, respectively. DSPN was diagnosed in 2.7%, and none met the criteria for probable DSPN. The prevalence of CAN and early CAN was 8.7% and 28.4%, respectively.

The characteristics of the study population are shown in **Table 1**.

### Dicarbonyls and advanced glycation end-products

The mean plasma level of MG, glyoxal, and 3-DG was 116.3 nmol/L (111.3; 127.6), 215.7 nmol/L (186.1; 253.4), and 57.8 nmol/L (54.3; 62.6), respectively.

**Figure 1:**
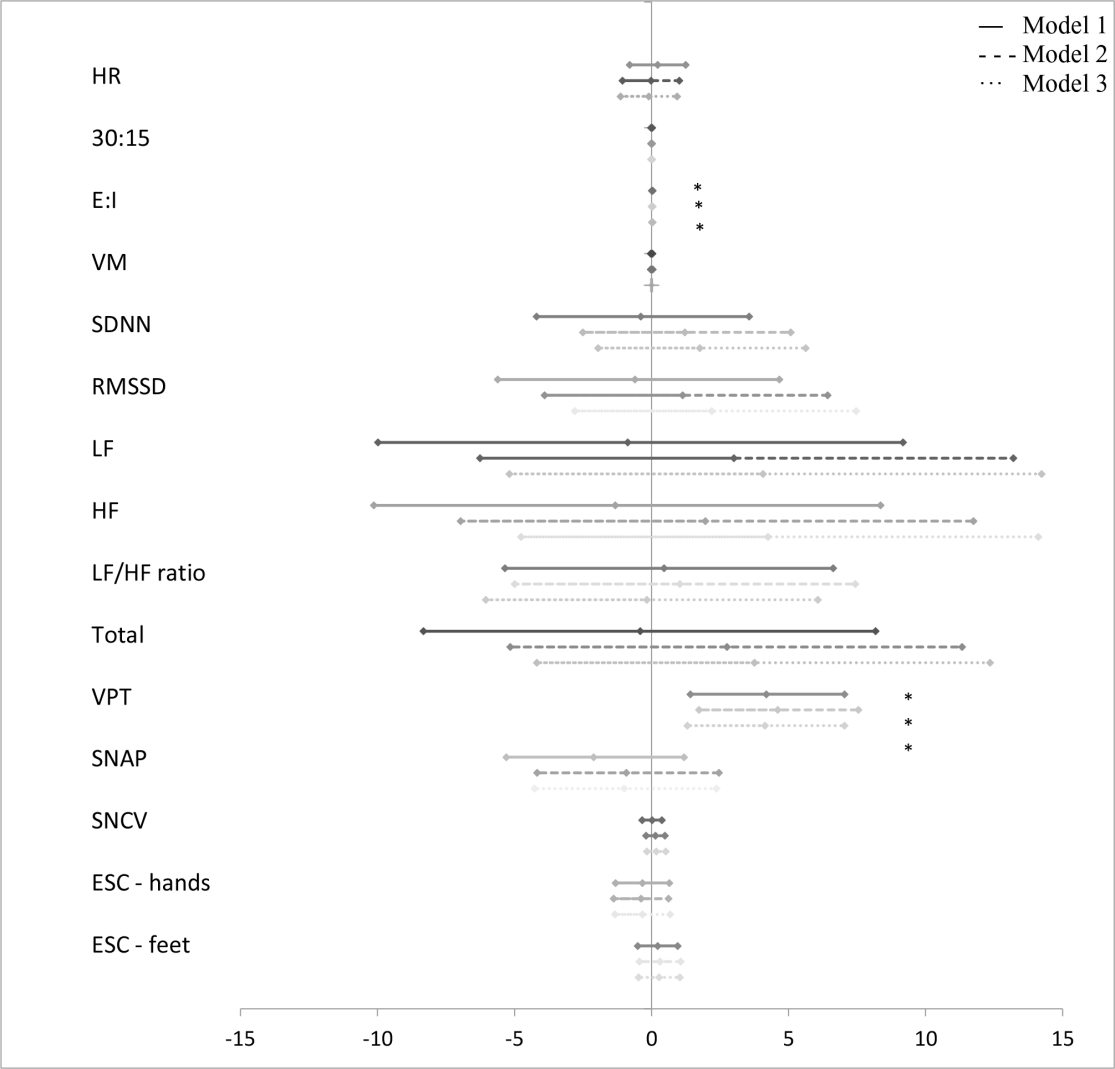
Forest plot of the associations between “glycolytic dysfunction” and measures of diabetic neuropathy. Results are presented as estimates and 95% confidence intervals. Estimates show the percentage change in the outcomes for an increase of one unit of “glycolytic dysfunction”. Studies with confidence interval crossing the vertical line are inconclusive. Model 1 adjusted for age and gender, model 2 adjusted as model 1 + diabetes duration and HbA_1c_, and model 3 adjusted as model 2 + current smoking, total cholesterol, triglycerides, systolic blood pressure, and the use of beta blockers. CAN, cardiovascular autonomic neuropathy; HR, heart rate; 30:15, lying-to-standing test; E:I, deep breathing test; VM, Valsalva Manoeuvre; SDNN, standard deviation of normal-to-normal intervals; RMSSD, root mean square of the sum of the squares of differences between consecutive R-R intervals; LF, low-frequency power; HF, high-frequency power; DSPN, distal symmetric polyneuropathy; VPT, vibration perception threshold; SNAP, sural nerve amplitude potential; SNCV, sural nerve conduction velocity; ESC, electrochemical skin conduction. *p < 0.05.

**Figure 2:**
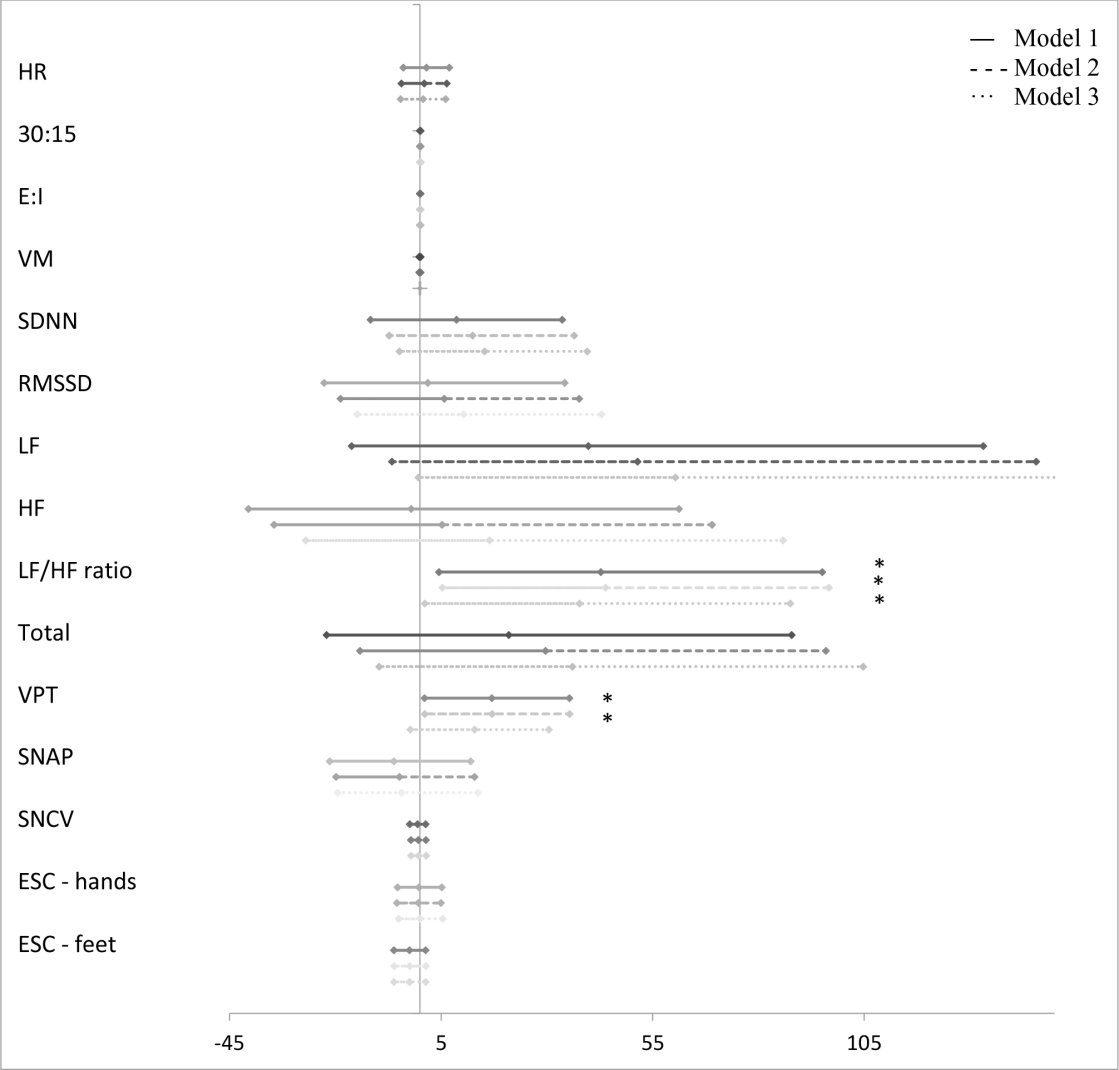
Forest plot of the associations between “lipid peroxidation” and measures of diabetic neuropathy. Results are presented as estimates and 95% confidence intervals. Estimates show the percentage change in the outcomes for an increase of one unit of “lipid peroxidation”. Studies with confidence interval crossing the vertical line are inconclusive. Model 1 adjusted for age and gender, model 2 adjusted as model 1 + diabetes duration and HbA_1c_, and model 3 adjusted as model 2 + current smoking, total cholesterol, triglycerides, systolic blood pressure, and the use of beta blockers. CAN, cardiovascular autonomic neuropathy; HR, heart rate; 30:15, lying-to-standing test; E:I, deep breathing test; VM, Valsalva Manoeuvre; SDNN, standard deviation of normal-to-normal intervals; RMSSD, root mean square of the sum of the squares of differences between consecutive R-R intervals; LF, low-frequency power; HF, high-frequency power; DSPN, distal symmetric polyneuropathy; VPT, vibration perception threshold; SNAP, sural nerve amplitude potential; SNCV, sural nerve conduction velocity; ESC, electrochemical skin conduction. *p < 0.05.

**Figure 3:**
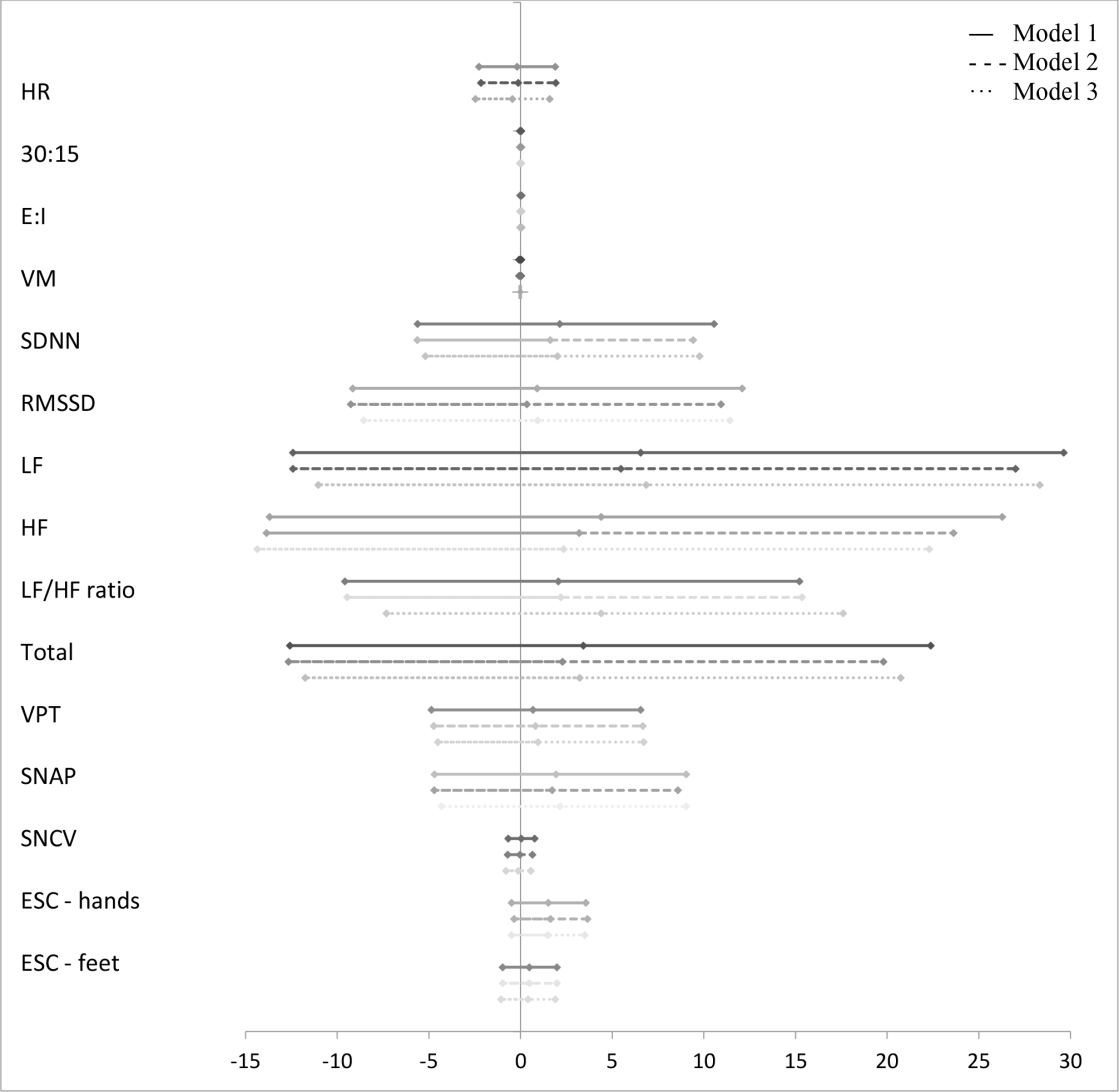
Forest plot of the associations between “glucotoxicity” and measures of diabetic neuropathy. Results are presented as estimates and 95% confidence intervals. Estimates show the percentage change in the outcomes for an increase of one unit of “glucotoxicity”. Studies with confidence interval crossing the vertical line are inconclusive. Model 1 adjusted for age and gender, model 2 adjusted as model 1 + diabetes duration and HbA_1c_, and model 3 adjusted as model 2 + current smoking, total cholesterol, triglycerides, systolic blood pressure, and the use of beta blockers. CAN, cardiovascular autonomic neuropathy; HR, heart rate; 30:15, lying-to-standing test; E:I, deep breathing test; VM, Valsalva Manoeuvre; SDNN, standard deviation of normal-to-normal intervals; RMSSD, root mean square of the sum of the squares of differences between consecutive R-R intervals; LF, low-frequency power; HF, high-frequency power; DSPN, distal symmetric polyneuropathy; VPT, vibration perception threshold; SNAP, sural nerve amplitude potential; SNCV, sural nerve conduction velocity; ESC, electrochemical skin conduction. *p < 0.05.

**Figure 4:**
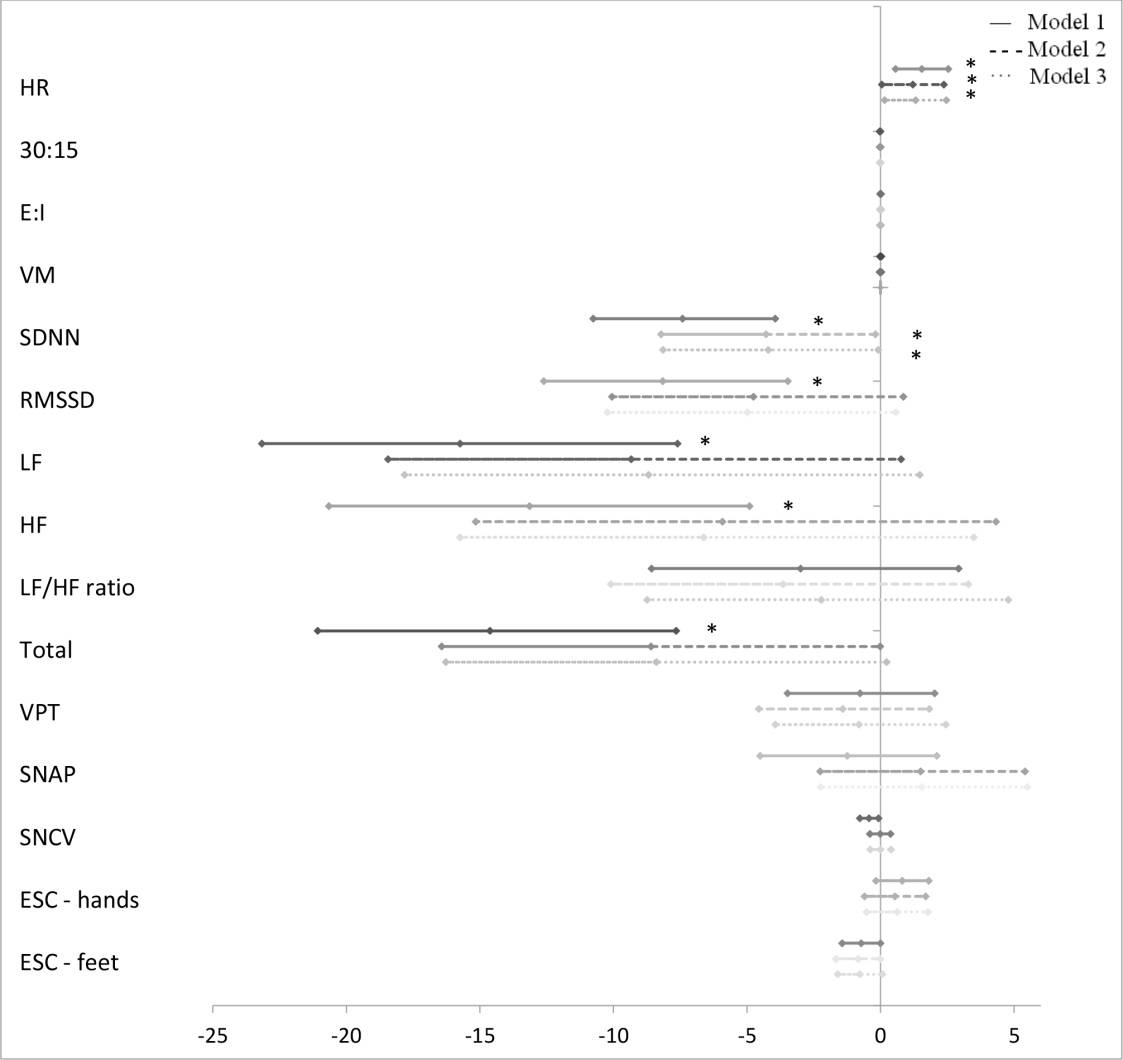
Forest plot of the associations between “dicarbonyls” and measures of diabetic neuropathy. Results are presented as estimates and 95% confidence intervals. Estimates show the percentage change in the outcomes for an increase of one unit of “dicarbonyls”. Studies with confidence interval crossing the vertical line are inconclusive. Model 1 adjusted for age and gender, model 2 adjusted as model 1 + diabetes duration and HbA_1c_, and model 3 adjusted as model 2 + current smoking, total cholesterol, triglycerides, systolic blood pressure, and the use of beta blockers. CAN, cardiovascular autonomic neuropathy; HR, heart rate; 30:15, lying-to-standing test; E:I, deep breathing test; VM, Valsalva Manoeuvre; SDNN, standard deviation of normal-to-normal intervals; RMSSD, root mean square of the sum of the squares of differences between consecutive R-R intervals; LF, low-frequency power; HF, high-frequency power; DSPN, distal symmetric polyneuropathy; VPT, vibration perception threshold; SNAP, sural nerve amplitude potential; SNCV, sural nerve conduction velocity; ESC, electrochemical skin conduction. *p < 0.05.

**Figure 5:**
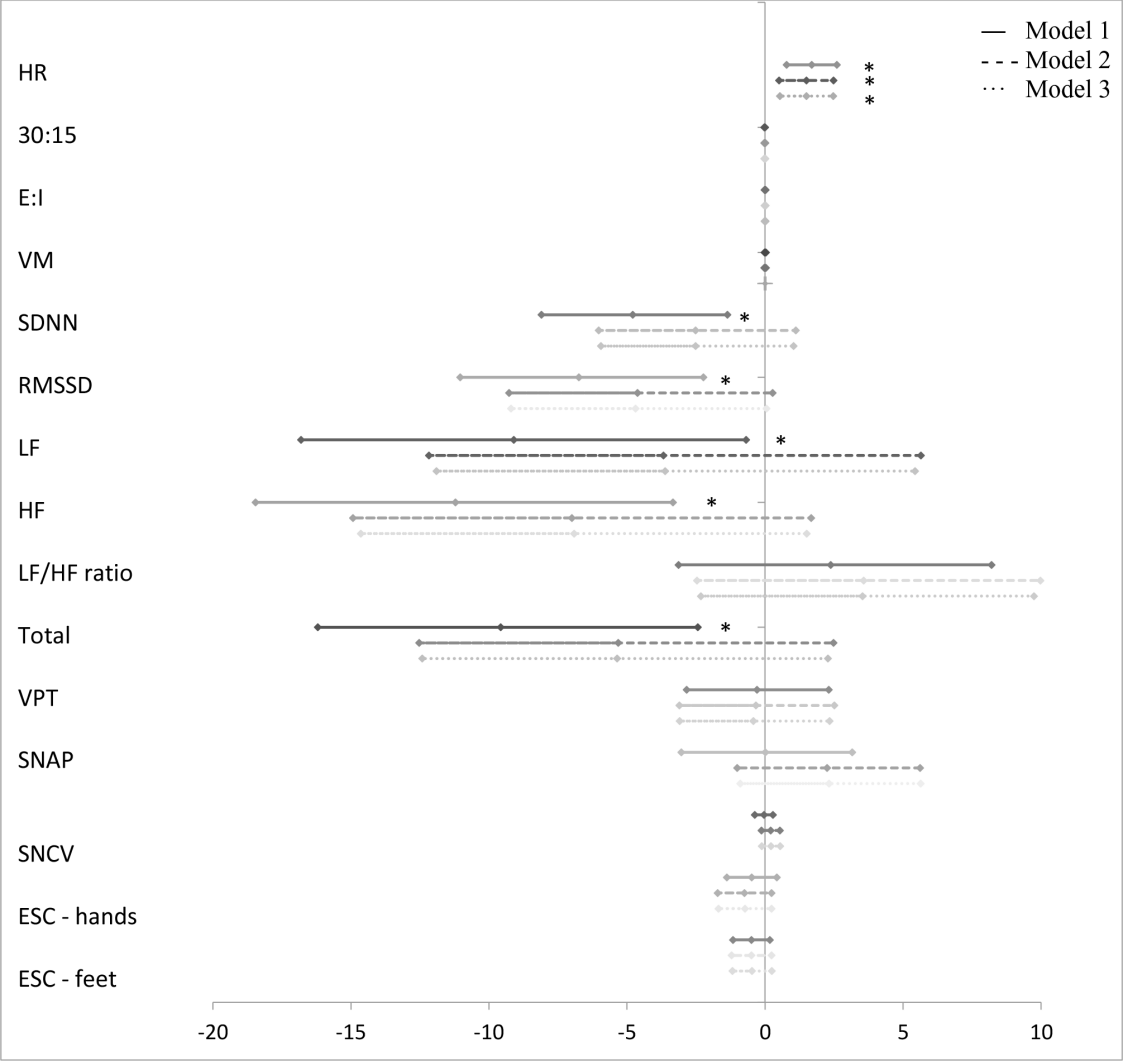
Forest plot of the associations between “oxidative stress” and measures of diabetic neuropathy. Results are presented as estimates and 95% confidence intervals. Estimates show the percentage change in the outcomes for an increase of one unit of “oxidative stress”. Studies with confidence interval crossing the vertical line are inconclusive. Model 1 adjusted for age and gender, model 2 adjusted as model 1 + diabetes duration and HbA_1c_, and model 3 adjusted as model 2 + current smoking, total cholesterol, triglycerides, systolic blood pressure, and the use of beta blockers. CAN, cardiovascular autonomic neuropathy; HR, heart rate; 30:15, lying-to-standing test; E:I, deep breathing test; VM, Valsalva Manoeuvre; SDNN, standard deviation of normal-to-normal intervals; RMSSD, root mean square of the sum of the squares of differences between consecutive R-R intervals; LF, low-frequency power; HF, high-frequency power; DSPN, distal symmetric polyneuropathy; VPT, vibration perception threshold; SNAP, sural nerve amplitude potential; SNCV, sural nerve conduction velocity; ESC, electrochemical skin conduction. *p < 0.05.

The associations between groups of AGEs and measures of CAN and DSPN are presented in **Figures 1**–**5** with forest plots as estimates and 95% CI.

### Cardiovascular autonomic neuropathy measures

A higher z-score of “glycolytic dysfunction” was associated with a higher E/I also when adjusted in model 3 (0.03% (95% CI 0.01; 0.05), p = 0.005) (**Figure 1**).

“Lipid peroxidation” was associated with a higher LF/HF ratio. Further adjustment in model 3 did not affect the association (37.72% (95% CI 1.12; 87.57), p = 0.044) (**Figure 2**).

A higher z-score of “glucotoxicity” was associated with lower values of the HRV indices SDNN, RMSSD, LF, HF, and total power in model 1 (**Figure 3**). Only for SDNN did significance remain after further adjustment in model 3 (−4.20% (95% CI −8.14; −0.09), p = 0.047). “Dicarbonyls” was also associated with lower values of the HRV indices. However, significance was lost in models 2 and 3 (**Figure 4**). In addition, higher z-scores of “glucotoxicity” and “dicarbonyls” were both associated with higher HR when fully adjusted in model 3 (1.31% (95% CI 0.16; 2.47), p = 0.027) and 1.51% (95% CI 0.54; 2.47), p = 0.003), respectively).

When applying the Benjamini–Hochberg procedure to account for multiple testing, all associations found between the groups of AGEs and dicarbonyls and measures of CAN lost significance.

No associations were found between measures of “oxidative stress” and any measures of cardiovascular autonomic neuropathy (**Figure 5**).

### Peripheral neuropathy measures

A higher z-score of “glycolytic dysfunction” was associated with a higher VPT in all three models (4.14% (95% CI 1.31; 7.04), p = 0.004) (**Figure 1**), but not when correcting for multiple tests. “Lipid peroxidation” was also associated with a higher VPT in models 1 and 2, but not when fully adjusted in model 3 (12.90% (95% CI −2.30; 30.45), p = 0.102) (**Figure 2**).

A higher z-score of “glucotoxicity” was associated with higher SNCV. However, the associations lost significance after adjustment in models 2 and 3 (**Figure 3**).

No associations were found between measures of peripheral neuropathy and “oxidative stress” or “dicarbonyls”.

## Discussion

In this cross-sectional study of 151 young adults with type 1 diabetes, we found that a number of pathways leading to AGE formation are activated in diabetic neuropathy. However, the relative contribution of glucose- and lipid-derived AGEs varied between the different measures of diabetic neuropathy, suggesting that both CAN and DSPN represent multiple symptoms involving more than one dysfunctional pathway. We found that increased levels of methylglyoxal- and glyoxal-derived AGEs representing changes in glycolytic function and lipid peroxidation, respectively, were associated with higher vibration perception thresholds. For CAN measures, we found that increased “glucotoxicity”, representing glucose-derived AGEs, was associated with higher HR and lower SDNN. “Dicarbonyls” was also associated with higher HR. However, after adjustments for multiple testing, no significant associations remained.

Previous studies in experimental models of diabetes as well as in diabetic humans have emphasized the importance of AGEs and their precursors, the reactive dicarbonyls, for the development of diabetic complications (13, 28). In diabetic neuropathy, disturbances in metabolic pathways caused by hyperglycaemia are believed to play a causative role. The higher glucose flux leads to increased formation of dicarbonyls mainly from glycolysis intermediates and lipid peroxidation (13). The formation and accumulation of reactive dicarbonyls are expected to be higher in neuronal tissue, as its primary source of energy is glucose (28). Moreover, increased formation of reactive oxygen species (ROS) leading to oxidative stress may contribute to neuronal dysfunction as ROS indirectly inhibits glyoxalase 1 (GLO-1) activity, which plays a role in the metabolization of dicarbonyl species (28).

In type 2 diabetes, studies have not presented uniform results when investigating the association between the reactive dicarbonyl, methylglyoxal, and diabetic neuropathy. Increased levels of plasma methylglyoxal have been associated with diabetic painful peripheral neuropathy by modifications of the voltage-gated nociceptor-specific sodium channel Nav1.8, which is associated with enhanced electrical excitability and facilitates firing of nociceptive neurons, thereby resulting in hyperalgesia (16). A recent prospective observational study of 1,256 new-onset type 2 diabetes patients found that higher levels of methylglyoxal are a risk factor for the development of diabetic polyneuropathy as assessed by the MNSI questionnaire (29). However, no association between serum methylglyoxal and diabetic autonomic and peripheral neuropathy was found in the cross-sectional study at the 6-year follow-up in the same cohort (30). Thus, increased methylglyoxal levels may play a causal role in painful neuropathy but not necessarily the other symptoms of diabetic neuropathy.

In our study, “dicarbonyls” were only associated with higher HR, but no other measures of CAN or DSPN. However, plasma levels of dicarbonyls might not represent dicarbonyl levels in nervous tissue, as it is shown in animal models that the activity of GLO-1 is low in nervous tissue compared to others (28). In addition, AGEs derived from methylglyoxal as well as other dicarbonyl species might play a major role. In line with this, we found that increased “lipid peroxidation”, although not statistically significant when fully adjusted, and “glycolytic dysfunction” representing glyoxal- and methylglyoxal-derived AGEs, respectively, were positively associated with worse vibration perception threshold. Previous clinical studies support the role of methylglyoxal-derived AGEs in diabetic neuropathy. A prospective cohort study of 216 humans with type 1 diabetes found that skin levels of AGEs were associated with the progression of diabetic complications including neuropathy (17, 19). The correlation of MG-H1 with diabetic neuropathy remained strongly associated when adjusted for all other risk factors. In addition, increased serum levels of MG-H1 have been associated with foot heat and pain detection threshold in patients with long-standing type 1 diabetes (18). Furthermore, increased serum levels of the glyoxal-derived AGE, CML, were associated with the development of small-fibre dysfunction. Despite studies supporting the role of methylglyoxal- and glyoxal-derived AGEs in diabetic neuropathy (17–19), it remains unknown which pathway is causing which symptom, and it does not explain why one symptom of neuropathy is more distinct than the others in patients with diabetes, as the studies do not discriminate the pathways involved; thus, the differences observed may be spurious.

In our study, “glucotoxicity” was inversely associated with several HRV indices, indicating autonomic dysfunction. However, most associations disappeared once adjusted for relevant confounders. Only a significant and consistent association between lower SDNN and higher resting HR remained. Also, “dicarbonyls” was associated with higher resting HR, while “lipid peroxidation” was associated with a higher LF/HF ratio. These findings support our hypothesis that a number of dysfunctional pathways are involved in CAN and implicate that the reactive metabolites are detrimental to the autonomic nervous system. However, all the associations were insignificant when adjusted for multiple testing.

Thus, our study indicates that AGE formation involving different dysfunctional pathways seems to play a diverse role in the pathogeneses of early diabetic neuropathy in young adults with type 1 diabetes in line with the relatively few previous studies in both type 1 and type 2 diabetes. This supports the hypothesis that metabolic derangements, rather than hyperglycaemia per se, may be the main cause contributing to diabetic complications.

## Strengths and limitations

A limitation of this study is the relatively low prevalence of definite CAN and DSPN in this population of young adults with type 1 diabetes, which might affect the ability to detect associations between AGEs and measures of neuropathy. However, objective measures of neuropathy assessed by novel and established methods of detecting CAN and DSPN have been applied, which is a strength of our study, as we have detected early stages of neuropathy. Further, we have grouped the AGEs with respect to their main precursors and thereby being able to specify potential pathways involved in the pathogeneses of neuropathy. Causal conclusions are, however, difficult to make due to the cross-sectional design of our study. The small sample size is another limitation and might affect the power of our study, thereby not making it generalizable. In addition, the significance of associations was lost when adjusting for multiple testing, indicating that the population size limited the power of analyses. Also, a healthy control group in our study would have enabled a comparison with age- and gender-matched non-diabetic young adults. However, this was not in the scheme of the study.

It is also recommended that participants avoid smoking, several drugs, and meals before the CARTs and HRV test, but we did not meet these recommendations, which may therefore affect CAN measures.

## Conclusion

In young adults with type 1 diabetes, we found that “glycolytic dysfunction”, “lipid peroxidation”, and “glucotoxicity”, all pathways leading to AGE formation, are involved in both CAN and DSPN. However, the involvement of the pathways varied between measures of CAN and DSPN, which reflects the diverse nature of both types of neuropathy. Thus, our study indicates that AGEs mainly derived from changes in glycolytic function and lipid peroxidation may contribute to decreased vibration sensation, while AGEs derived from glucose are related to autonomic dysfunction.

Despite the lost associations after adjustments for multiple testing, our results indicate a possible association between some AGEs and different measures of neuropathy in a relatively young population with a modest prevalence of neuropathy. This suggests that AGEs even in the early stages of diabetes may play a diverse role in the pathogeneses of different types of diabetic neuropathy.

## Data availability statement

The raw data supporting the conclusions of this article will be made available by the authors, without undue reservation.

## Ethics statement

This study was reviewed and approved by The Danish Research Ethics Committee (Id. No.:H-15006967). The patients/participants provided their written informed consent to participate in this study.

## Author contributions

EA-S analysed and interpreted the data and drafted the article. MC contributed to the design of the study, acquired and interpreted data, drafted the article, and approved the final version to be published. PN analysed blood samples for AGEs, interpreted data, revised the article critically, and approved the final version to be published. TF analysed blood samples for AGEs, interpreted data, revised the article critically, and approved the final version to be published. EH contributed to the design of the study, analysed and interpreted data, revised the article critically, and approved the final version to be published. MJ contributed to the design of the study, analysed and interpreted data, revised the article critically, and approved the final version to be published. JF contributed to the design of the study, analysed and interpreted data, revised the article critically, and approved the final version to be published. CH contributed to the design of the study and acquisition, analysis, and interpretation of data. He revised the article critically and approved the final version to be published. All authors contributed to the article and approved the submitted version.

## Funding

This study was supported by the Deutsche Forschungsgemeinschaft (SFB 1118 and GRK 1874-DIAMICOM), The Augustinus Foundation (15-2274), and The Toyota Foundation (BG 8687).

## Acknowledgments

EA-S is the guarantor of this work and, as such, had full access to all the data in the study and takes responsibility for the integrity of the data and the accuracy of the data analysis.

## Conflict of interest

JF holds stocks in Medicus Engineering. EH, MC and MJ hold shares in Novo Nordisk AS. MJ has received research grants from AMGEN, Astra Zeneca, Boehringer Ingelheim, Novo Nordisk, and Sanofi Aventis.

The remaining authors declare that the research was conducted in the absence of any commercial or financial relationships that could be construed as a potential conflict of interest.

## Publisher’s note

All claims expressed in this article are solely those of the authors and do not necessarily represent those of their affiliated organizations, or those of the publisher, the editors and the reviewers. Any product that may be evaluated in this article, or claim that may be made by its manufacturer, is not guaranteed or endorsed by the publisher.
