# Supplementary material for: Advanced glycation end-products are associated with diabetic neuropathy in young adults with type 1 diabetes
Source: Front Endocrinol (Lausanne). 2022 Oct 11;13:891442. doi: 10.3389/fendo.2022.891442 (PMC9592972; doi:10.3389/fendo.2022.891442)
Supplement: Supplementary file 1 [file Table_1.docx]

**Supplementary materials
Table 1** The association between “glycolytic dysfunction” and measures of diabetic neuropathy.

|  | Model 1 | Model 2 | Model 3 |
| --- | --- | --- | --- |
|  |  |  |  |
| **CAN measures**  Heart rate  Lying to standing (30:15)  Deep breathing (E/I)  Valsalva Maneuver (VM)  SDNN  RMSSD  LF  HF  LF/HF ratio  Total  **DSPN Measures**  VPT  SNAP  SNCV  ESC – hands  ESC - feet | 0.23 (-0.80;1.25) -0.00 (-0.02:0.02) 0.03 (0.01;0.04)* 0.00 ( -0.03;0.03) -0.39 (-4.19;3.56)  -0.60 (-5.61;4.67) -0.86 (-9.98;9.18) -1.32 (-10.13;8.36)  0.46 (-5.35;6.63) -0.42 (-8.33:8.18)  4.19 (1.41;7.04)* -2.11 (-5.30;1.18) 0.02 (-0.34;0.38) -0.34 (-1.31;0.65) 0.22 (-0.50;0.95) | **Estimate (95% CI)** -0.02 (-1.06;1.02) 0.00 (-0.02;0.02) 0.03 (0.01;0.04)* 0.00 ( -0.03;0.03)  1.22 (-2.51;5.08) 1.13 (-3.90;6.42) 3.01 (-6.26;13.20) 1.96 (-6.97;11.75) 1.03 (-4.99;7.44) 2.76 (-5.16;11.33)  4.60 (1.73;7.55)* -0.92 (-4.18;2.45) 0.14 (-0.20;0.49)  -0.39 (-1.39;0.62) 0.30 (-0.45;1.06) | -0.10 (-1.13;0.93) 0.00 (-0.02;0.02) 0.03 (0.01;0.05)* 0.00 (-0.02;0.03) 1.77 (-1.95;5.62) 2.20 (-2.80;7.46) 4.07 (-5.19;14.23) 4.25 (-4.76;14.11) -0.17 (-6.05; 6.07) 3.76 (-4.19;12.36)  4.14 (1.31;7.04)* 1.00 (-4.26;2.37) 0.18 (-0.17;0.52) -0.33 (-1.33;0.68) 0.28 (-0.47;1.03) |
| *Results are presented as estimates. Estimates show the percentage change in the outcomes for every 1-unit change of “glycolytic dysfunction”* ((% change (95% CI)). *Model 1 adjusted for age and gender, model 2 adjusted as model 1 + diabetes duration and HbA_1c_, model 3 adjusted as model 2 + current smoking, total cholesterol, triglycerides, systolic blood pressure and the use of beta blockers. CAN, cardiovascular autonomic neuropathy; HR, heart rate; 30:15, lying-to-standing test; E:I, deep breathing test; VM, Valsalva Manoeuvre; SDNN, standard deviation of normal-to-normal intervals; RMSSD, root mean square of the sum of the squares of differences between consecutive R-R intervals; LF, low-frequency power; HF, high-frequency power; DSPN, distal symmetric polyneuropathy; VPT, vibration perception threshold; SNAP, sural nerve amplitude potential; SNCV, sural nerve conduction velocity; ESC, electrochemical skin conduction. *P < 0.05.* | | | |
