# Supplementary material for: Advanced glycation end-products are associated with diabetic neuropathy in young adults with type 1 diabetes
Source: Front Endocrinol (Lausanne). 2022 Oct 11;13:891442. doi: 10.3389/fendo.2022.891442 (PMC9592972; doi:10.3389/fendo.2022.891442)
Supplement: Supplementary file 2 [file Table_2.docx]

**Table 2** The association between “lipid peroxidation” and measures of diabetic neuropathy.

|  | Model 1 | Model 2 | Model 3 |
| --- | --- | --- | --- |
| **CAN Measures** |  | **Estimate (95% CI)** |  |
| Heart rate  Lying to standing (30:15)  Deep breathing (E/I)  Valsalva Maneuver (VM)  SDNN  RMSSD  LF  HF  LF/HF ratio  Total  **DSPN Measures**  VPT  SNAP  SNCV  ESC – hands  ESC - feet | 1.51 (-3.93;6.96) 0.07 (-0.03;0.16) 0.05 (-0.05;0.15) -0.00 (-0.15;0.15) 8.63 (-11.68;33.62)  1.90 (-22.63;34.21) 39.83 (-16.16;133.19) -2.08 (-40.53;61.25) 42.79 (4.50;95.13)*  21.00 (-22.06;87.87)  16.97 (1.11;35.32)* -6.12 (-21.32;12.02)  -0.53 (-2.43;1.36) -0.20 (-5.30;5.18) -2.46 (-6.13;1.35) | 1.00 (-4.35;6.35) 0.07 (-0.02;0.17) 0.05 (-0.05;0.15) -0.01 (-0.16;0.14) 12.47 (-7.30;36.45)  5.77 (-18.74;37.67) 51.45 (-6.63;145.64)  5.24 (-34.50;69.08) 43.92 (5.30;96.70)* 29.71 (-14.14;95.97)  17.04 (1.16;35.41)* -4.85 (-19.83;12.92)  -0.36 (-2.12;1.40) -0.36 (-5.40;4.95)  -2.39 (-6.06;1.43) | 0.74 (-4.57;6.06) 0.08 (-0.02;0.17)  0.06 (-0.04;0.15) -0.01 (-0.16;0.14) 15.27 (-4.80;39.58) 10.35 (-14.84;42.99  60.41 (-0.33;158.18) 16.48 (-27.00;85.86) 37.72 (1.12;87.57)* 36.05 (-9.62;104.78)  12.90 (-2.30;30.45)  -4.28 (-19.41;13.69) -0.28 (-2.05;1.50) 0.01 (-5.06;5.35) -2.48 (-6.16;1.33) |
| *Results are presented as estimates. Estimates show the percentage change in the outcomes for every 1-unit change of “lipid peroxidation”* ((% change (95% CI)). *Model 1 adjusted for age and gender, model 2 adjusted as model 1 + diabetes duration and HbA_1c_, model 3 adjusted as model 2 + current smoking, total cholesterol, triglycerides, systolic blood pressure and the use of beta blockers. CAN, cardiovascular autonomic neuropathy; HR, heart rate; 30:15, lying-to-standing test; E:I, deep breathing test; VM, Valsalva Manoeuvre; SDNN, standard deviation of normal-to-normal intervals; RMSSD, root mean square of the sum of the squares of differences between consecutive R-R intervals; LF, low-frequency power; HF, high-frequency power; DSPN, distal symmetric polyneuropathy; VPT, vibration perception threshold; SNAP, sural nerve amplitude potential; SNCV, sural nerve conduction velocity; ESC, electrochemical skin conduction. *P < 0.05.* | | | |
