# Supplementary material for: Advanced glycation end-products are associated with diabetic neuropathy in young adults with type 1 diabetes
Source: Front Endocrinol (Lausanne). 2022 Oct 11;13:891442. doi: 10.3389/fendo.2022.891442 (PMC9592972; doi:10.3389/fendo.2022.891442)
Supplement: Supplementary file 3 [file Table_3.docx]

**Table 3** The association between “oxidative stress” and measures of diabetic neuropathy.

|  | Model 1 | Model 2 | Model 3 |
| --- | --- | --- | --- |
| **CAN Measures** |  | **Estimate (95% CI)** |  |
| Heart rate  Lying to standing (30:15)  Deep breathing (E/I)  Valsalva Maneuver (VM)  SDNN  RMSSD  LF  HF  LF/HF ratio  Total  **DSPN Measures**  VPT  SNAP  SNCV  ESC – hands  ESC - feet | -0.19 (-2.27;1.89) 0.01 (-0.03:0.04) 0.02 (-0.0141;0.06) -0.02 (-0.08;0.04) 2.14 (-5.63;10.55) 0.91 (-9.16;12.09) 6.56 (-12.41;29.63) 4.40 (-13.68;26.27) 2.07 (-9.58;15.21) 3.43 (-12.58;22.38)  0.68 (-4.86;6.55)  1.94 (-4.70;9.04) 0.05 (-0.68;0.77)  1.52 (-0.49;3.56)  0.49 (-0.98;1.98) | -0.12 (-2.16;1.92) 0.01 (-0.03:0.04) 0.02 (-0.02;0.06) -0.02 (-0.08;0.04) 1.61 (-5.629;9.41)  0.34 (-9.25;10.94) 5.47 (-12.42;27.01) 3.20 (-13.85;23.63) 2.20 (-9.45;15.36) 2.29 (-12.66;19.79)   0.81 (-4.74;6.68) 1.73 (-4.71;8.59) -0.03 (-0.70;0.64)  1.64 (-0.35;3.66) 0.49 (-0.98;1.98) | -0.44 (-2.46;1.59) 0.01 (-0.03;0.04) 0.02 (-0.01;0.06)   -0.02 (-0.08;0.04) 2.01 (-5.19;9.77) 0.93 (-8.57;11.41) 6.85 (-11.04;28.33)  2.34 (-14.35;22.30) 4.40 (-7.32;17.60) 3.22 (-11.75;20.74)  0.95 (-4.52;6.72) 2.14 (-4.32;9.04)  -0.12 (-0.80;0.55)  1.49 (-0.49;3.51) 0.41 (-1.06;1.89) |
| *Results are presented as estimates. Estimates show the percentage change in the outcomes for every 1-unit change of “oxidative stress”* ((% change (95% CI)). *Model 1 adjusted for age and gender, model 2 adjusted as model 1 + diabetes duration and HbA_1c_, model 3 adjusted as model 2 + current smoking, total cholesterol, triglycerides, systolic blood pressure and the use of beta blockers. CAN, cardiovascular autonomic neuropathy; HR, heart rate; 30:15, lying-to-standing test; E:I, deep breathing test; VM, Valsalva Manoeuvre; SDNN, standard deviation of normal-to-normal intervals; RMSSD, root mean square of the sum of the squares of differences between consecutive R-R intervals; LF, low-frequency power; HF, high-frequency power; DSPN, distal symmetric polyneuropathy; VPT, vibration perception threshold; SNAP, sural nerve amplitude potential; SNCV, sural nerve conduction velocity; ESC, electrochemical skin conduction. *P < 0.05.* | | | |
