# Supplementary material for: Advanced glycation end-products are associated with diabetic neuropathy in young adults with type 1 diabetes
Source: Front Endocrinol (Lausanne). 2022 Oct 11;13:891442. doi: 10.3389/fendo.2022.891442 (PMC9592972; doi:10.3389/fendo.2022.891442)
Supplement: Supplementary file 4 [file Table_4.docx]

**Table 4** The association between “glucotoxicity” and measures of diabetic neuropathy.

|  | Model 1 | Model 2 | Model 3 |
| --- | --- | --- | --- |
| **CAN Measures** |  | **Estimate (95% CI)** |  |
| Heart rate  Lying to standing (30:15)  Deep breathing (E/I)  Valsalva Maneuver (VM)  SDNN  RMSSD  LF  HF  LF/HF ratio  Total  **DSPN Measures**  VPT  SNAP  SNCV  ESC – hands  ESC – feet | 1.55 (0.56;2.54)* -0.02 (-0.04;-0.00)* 0.00 (-0.02;0.02) 0.01 (-0.02;0.03) -7.41 (-10.76;-3.94)* -8.15 (-12.60;-3.47)* -15.74 (-23.16;-7.60)* -13.14 (-20.66;-4.90)* -3.00 (-8.57;2.92) -14.62 (-21.07;-7.65)*  -0.76 (-3.48;2.03) -1.25 (-4.50;2.11) -0.43 (-0.78;-0.07)*  0.81 (-0.17;1.80) -0.72 (-1.44;0.00) | 1.21 (0.06;2.37)* -0.01 (-0.03;0.01) 0.00 (-0.02;0.03) -0.00 (-0.03;0.03) -4.29 (-8.22;-0.19)* -4.75 (-10.05;0.86) -9.34 (-18.44;0.77) -5.92 (-15.16;4.33) -3.64 (-10.09;3.29) -8.59 (-16.43;-0.01)  -1.42 (-4.56;1.83) 1.50 (-2.26;5.41) -0.01 (-0.40;0.38)  0.54 (-0.60;1.69) -0.83 (-1.66;-0.00) | 1.31 (0.16;2.47)* -0.01 (-0.03;0.01)  0.00 (-0.02;0.02) -0.00 (-0.03;0.03) -4.20 (-8.14;-0.09)* -4.98 ( -10.23;0.57)  -8.69 (-17.83;1.47)  -6.62 (-15.74;3.49) -2.21 (-8.74;4.78) -8.40 (-16.28;0.23)  -0.80 (-3.94;2.44) 1.55 (-2.24;5.49)  0.00 (-0.39;0.39) 0.62 (-0.52;1.78) -0.77 (-1.60;0.07) |
| *Results are presented as estimates. Estimates show the percentage change in the outcomes for every 1-unit change of “glucotoxicity”* ((% change (95% CI)). *Model 1 adjusted for age and gender, model 2 adjusted as model 1 + diabetes duration and HbA_1c_, model 3 adjusted as model 2 + current smoking, total cholesterol, triglycerides, systolic blood pressure and the use of beta blockers. CAN, cardiovascular autonomic neuropathy; HR, heart rate; 30:15, lying-to-standing test; E:I, deep breathing test; VM, Valsalva Manoeuvre; SDNN, standard deviation of normal-to-normal intervals; RMSSD, root mean square of the sum of the squares of differences between consecutive R-R intervals; LF, low-frequency power; HF, high-frequency power; DSPN, distal symmetric polyneuropathy; VPT, vibration perception threshold; SNAP, sural nerve amplitude potential; SNCV, sural nerve conduction velocity; ESC, electrochemical skin conduction. *P < 0.05.* | | | |
