# Supplementary material for: Advanced glycation end-products are associated with diabetic neuropathy in young adults with type 1 diabetes
Source: Front Endocrinol (Lausanne). 2022 Oct 11;13:891442. doi: 10.3389/fendo.2022.891442 (PMC9592972; doi:10.3389/fendo.2022.891442)
Supplement: Supplementary file 5 [file Table_5.docx]

**Table 5** The association between “dicarbonyls” and measures of diabetic neuropathy.

|  | Model 1 | Model 2 | Model 3 |
| --- | --- | --- | --- |
| **CAN Measures** |  | **Estimate (95% CI)** |  |
| Heart rate  Lying to standing (30:15)  Deep breathing (E/I)  Valsalva Maneuver (VM)  SDNN  RMSSD  LF  HF  LF/HF ratio  Total  **DSPN Measures**  VPT  SNAP  SNCV  ESC – hands  ESC - feet | 1.69 (0.78 ; 2.61)* -0.01 (-0.03; 0.00) 0.01 (-0.01;0.02) 0.01 (-0.02; 0.03) -4.79 (-8.10; -1.36)* -6.74 (-11.04; -2.23)* -9.10 (-16.80; -0.68)* -11.21 (-18.45;-3.33)* 2.38 (-3.14; 8.21) -9.60 (-16.20 ; -2.44)*  -0.29 (-2.83; 2.31) 0.02 (-3.03; 3.16)  -0.04 (-0.37; 0.29) -0.48 (-1.39; 0.43) -0.50 (-1.16; 0.17) | 1.50 (0.51; 2.48)* -0.01 (-0.03; 0.01) 0.00 (-0.01;0.02) 0.00 (-0.02: 0.03) -2.52 (-6.02; 1.11) -4.62 (-9.28; 0.28) -3.67 (-12.17; 5.65) -7.00 (-14.93; 1.67) 3.57 (-2.46; 9.98) -5.32 (-12.53; 2.48)  -0.33 (-3.10; 2.52) 2.25 (-1.01; 5.61) 0.21 (-0.12; 0.54)  -0.74 (-1.71; 0.24) -0.49 (-1.21; 0.24) | 1.51 (0.54; 2.47)* -0.01 (-0.03; 0.01)  0.00 (-0.01;0.02) 0.00 (-0.02: 0.03) -2.52 (-5.94; 1.03) -4.69 (-9.20; 0.06)  -3.62 (-11.90; 5.44) -6.92 (-14.64; 1.51) 3.54 (-2.32; 9.75) -5.35 (-12.41; 2.28)  -0.42 (-3.10; 2.34) 2.32 (-0.89; 5.64) 0.21 (-0.11; 0.55)  -0.73 (-1.68; 0.24) -0.47 (-1.18; 0.25) |
| *Results are presented as estimates. Estimates show the percentage change in the outcomes for every 1-unit change of “dicarbonyls”* ((% change (95% CI)). *Model 1 adjusted for age and gender, model 2 adjusted as model 1 + diabetes duration and HbA_1c_, model 3 adjusted as model 2 + current smoking, total cholesterol, triglycerides, systolic blood pressure and the use of beta blockers. CAN, cardiovascular autonomic neuropathy;* *HR, heart rate; 30:15, lying-to-standing test; E:I, deep breathing test; VM, Valsalva Manoeuvre; SDNN, standard deviation of normal-to-normal intervals; RMSSD, root mean square of the sum of the squares of differences between consecutive R-R intervals; LF, low-frequency power; HF, high-frequency power; DSPN, distal symmetric polyneuropathy; VPT, vibration perception threshold; SNAP, sural nerve amplitude potential; SNCV, sural nerve conduction velocity; ESC, electrochemical skin conduction. *P < 0.05.* | | | |
